# Imputation-Based Variable Selection Method for Block-Wise Missing Data When Integrating Multiple Longitudinal Studies

**DOI:** 10.3390/math12070951

**Published:** 2024-03-23

**Authors:** Zhongzhe Ouyang, Lu Wang, Alzheimer’s Disease Neuroimaging Initiative

**Affiliations:** Department of Biostatistics, University of Michigan, Ann Arbor, MI 48109, USA

**Keywords:** multiple imputation, correlated data, data integration, 62H99

## Abstract

When integrating data from multiple sources, a common challenge is block-wise missing. Most existing methods address this issue only in cross-sectional studies. In this paper, we propose a method for variable selection when combining datasets from multiple sources in longitudinal studies. To account for block-wise missing in covariates, we impute the missing values multiple times based on combinations of samples from different missing pattern and predictors from different data sources. We then use these imputed data to construct estimating equations, and aggregate the information across subjects and sources with the generalized method of moments. We employ the smoothly clipped absolute deviation penalty in variable selection and use the extended Bayesian Information Criterion criteria for tuning parameter selection. We establish the asymptotic properties of the proposed estimator, and demonstrate the superior performance of the proposed method through numerical experiments. Furthermore, we apply the proposed method in the Alzheimer’s Disease Neuroimaging Initiative study to identify sensitive early-stage biomarkers of Alzheimer’s Disease, which is crucial for early disease detection and personalized treatment.

## 1. Introduction

Multi-sources data are now attracting more attention in scientific research. A practical problem with multi-source data is block-wise missing. Our work is motivated by the existence of block-wise missingness in Alzheimer’s Disease Neuroimaging Initiative (ADNI) data when investigating the biomarkers that are associated with Alzheimer’s Disease (AD). In the ADNI study, healthy elderly subjects, as well as subjects with normal cognition (NC), mild cognitive impairment (MCI), or AD, were recruited to identify neuroimaging measures, cognitive measures and biomarkers that can effectively and timely detect cognitive and functional changes [1]. The ADNI data exhibit a block-wise missing structure along with the long duration of the study, and the high cost of certain measurements, etc. Besides the ADNI data, datasets with block-wise missing structure also exist across many other fields including environmental science, sociology, and economics. For example, a block-wise missing structure appears in human mortality data integrated from Italy and Switzerland [2] and in credit data collected from various institutions (Lan and Jiang [3]; Li et al. [4]).

Statistical analysis with missing covariates has been widely studied due to the prevalence of missing values in many datasets. Common methods for dealing with missing data include complete case analysis, maximum likelihood, inverse probability weighting, and imputation. While complete case analysis is the easiest approach to implement, it has several drawbacks, such as potential bias in certain cases and a significant loss of information when the proportion of missingness is high. The maximum likelihood approach (e.g., Sabbe et al. [5]; Bondarenko and Raghunathan [6]; Audigier et al. [7]; von Hippel and Bartlett [8]) requires a specification on the distribution of variables, though this is unknown and unverifiable in practice. Inverse probability weighting (e.g., Chen et al. [9]; Creemers et al. [10]; Zubizarreta [11]; Hughes et al. [12]) heavily relies on the information from complete cases, which can be problematic when the fraction of completely observed subject is small.

Two big challenges with the above ADNI data are the high proportion of missingness and the large number of covariates, which make the complete case analysis and maximum likelihood approach inefficient. In addition to these two challenges, weighted methods cannot handle the problem in presence of multiple missing patterns. Compared to these methods with notable limitations, imputation methods are more appropriate for the ADNI data. Recently, multi-source data with block-wise missing, exemplified by the ADNI data, have drawn extensively attention in statistically research. Ref. [13] developed a classification framework, which was accomplished by three steps: feature selection, sample selection, and matrix completion. Ref. [2] proposed a dimension reduction method called generalized integrative principal component analysis (GIPCA). Under the assumption of identical type of distribution in the exponential family within each data source, GIPCA decomposed the overall effect into joint and individual effect across data sources. Ref. [14] imputed the missing data using a factor structure model, which considered the correlation between predictors and does not depend on missing mechanism. Ref. [15] developed a multiple block-wise imputation (MBI) approach by constructing estimating functions based on both complete and incomplete observation. Other related literature include those of [4,16,17].

However, these methods are not applicable to longitudinal studies. Using these methods on the ADNI data, they only select baseline measurement for each patient and simply delete the following measurements. Thus, these methods are inefficient for the ADNI data since they fail to take account of with-subject correlations. In this paper, we aim to develop a method for variable selection when integrating longitudinal data from multiple sources in the existing block-wise missing structure. We impute the block-wise missing data multiple times by using the information from both subjects with complete observation and subjects with missing values. We construct estimating equations based on imputed data and incorporate working correlation matrices to account for within-cluster correlation. With the generalized method of moment, we are capable of integrating data from multiple sources and identifying the relevant variables by introducing a penalty term.

This paper is organized as follows. Section 2 describes the setup and formalize the proposed method. In Section 3, we study the asymptotic properties of the proposed estimator. In Section 4, we develop an algorithm to implement the proposed method, followed by a simulation study conducted in Section 5 to evaluate the performance of the proposed method. In Section 6, we apply the proposed method to the ADNI study. Section 7 provides further discussions.

## 2. Methods

### 2.1. Setup

Suppose the dataset consists of n independent and identically distributed (*i*.*i*.*d*.) samples drawn from independent sources with disjoint covariates. Without loss of generality, we assume that the data are already sorted by missing patterns, and the total number of missing patterns is K with $n_{k}$ samples in each pattern, where $\sum_{k=1}^{K}\;n_{k}=n$ and k=1,…,K. Within each missing pattern, all subjects have the same missing structure and the covariates from any specific source are either fully observed or fully missing. Let $Y_{k,i}={\left(Y_{k,i1},\ldots ,Y_{k,im_{i}}\right)}^{T}$ be the response vector for the *i*th subject in the *k*th pattern with $m_{i}$ measurements. For ease of presentation, we assume that each sample has the same number of measurements m. Furthermore, let $X_{k,i}=\left(X_{k,i1},\ldots ,X_{k,ip}\right)$ be the corresponding covariate matrix for the *i*th subject in the *k*th pattern across all the measurements, where p is the number of covariates. We assume the underlying population-level model is as follows:

$$E\left(Y_{k,i}|X_{k,i}\right)=\mu \left(X_{k,i}\beta \right),\;k=1,\ldots ,K,$$

where μ(⋅) is a known monotonic link function and β is a p-dimentional vector in the parameter space. Let O(k) and M(k) denote the index of observed covariates and missing covariates in the *k*th pattern, respectively. Define $R_{i}=1$ if $X_{k,i}$ is fully observed, otherwise 0. We assume the missing mechanism of $X_{k,i}$ is missing completely at random [18].

**Figure 1.**
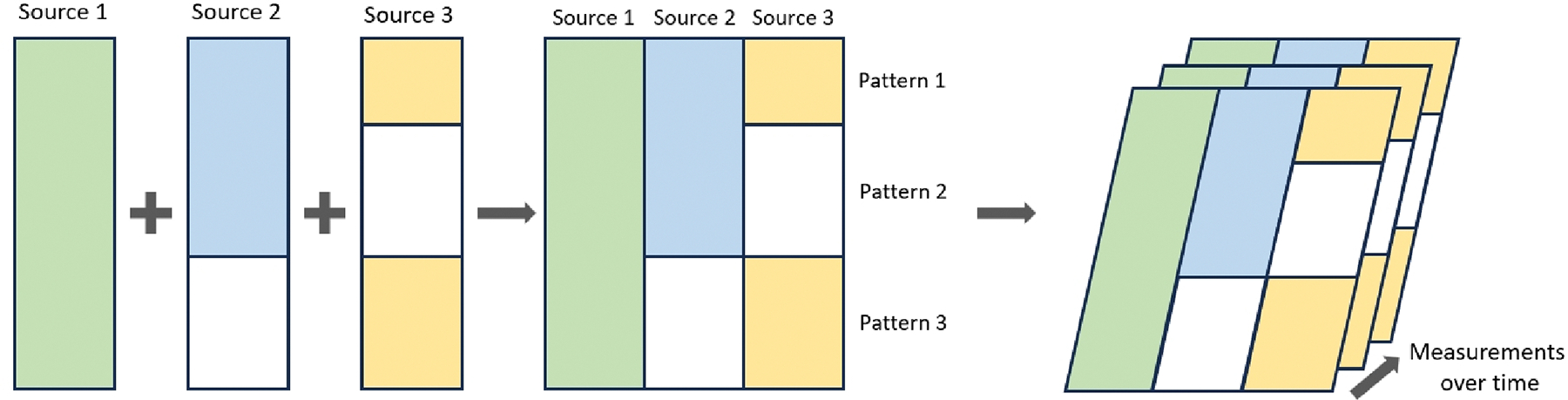
Example of block-wise missing data in longitudinal studies.

Figure 1 is an example illustrated what block-wise missing data look like, which consist of three sources with three missing patterns. Note that covariates in source 1 are completely observed in all three patterns, while covariates in source 2 are only observed in pattern 1 and 2, and covariates in source 3 are only observed in pattern 1 and 3. A similar structure also exists in the ADNI data. For example, variables in cerebrospinal fluid (CSF) are only measured in a subsample since CSF collection were mainly performed in phase II. Although complete cases analysis is feasible for ADNI data, it is inefficient especially the number of subjects with complete observation is limited. Thus, it is essential to leverage information from incomplete observation.

### 2.2. Proposed Method

**Figure 2.**
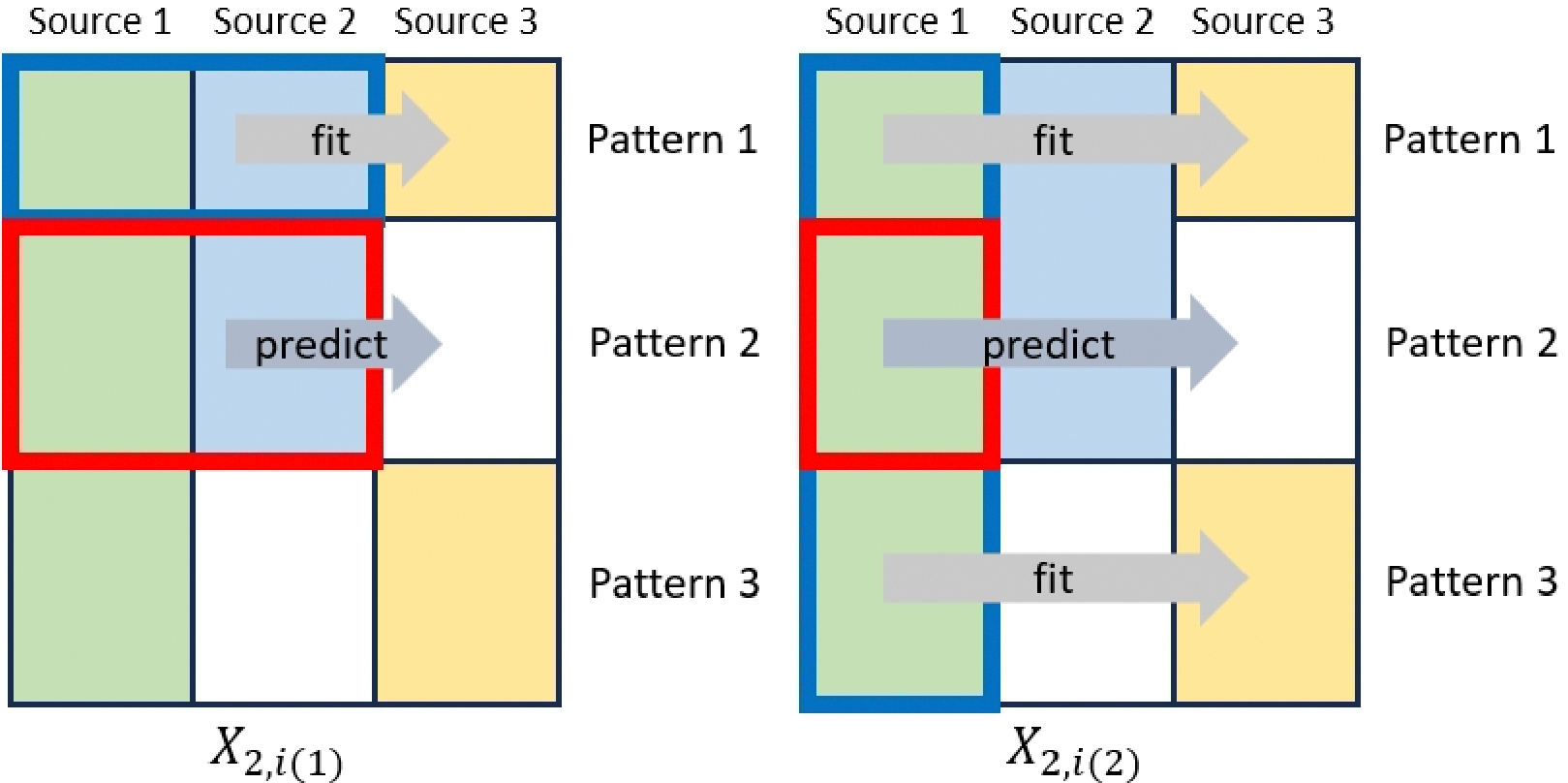
Two imputation approaches for missing covariates of source 3 in pattern 2. In the left figure, samples from pattern 1 and covariates in source 1 and source 2 are used to train the model, which is subsequently used to predict the missing covariates in pattern 2. Similarly, in the right figure, samples from pattern 1 and pattern 3 and covariates in source 1 are used to train the model.

One approach to utilizing incomplete data is by imputing missing values and performing statistical analysis based on the imputed dataset. Traditional methods impute missing values using information solely from complete cases. However, in scenarios involving block-wise missing data, the proportion of complete cases can be relatively small, resulting in unstable imputed values. To further illustrate how to incorporate information from subjects with partially observed values when imputing missing values, we continue to use the example given in Figure 1. Let $X_{k,i(r)}$ be the *r*th imputed covariate vector for the *i*th subject from pattern $k,r=1,\ldots ,R_{k}$. For instance, the missing values of $X_{2,i}$, i.e., the covariates of source 3 in pattern 2, can be imputed using the information of all sources in pattern 1, which we denoted as $X_{2,i(1)}$. Additionally, these can also be imputed based on the covariates in source 1 and source 3 for subjects from pattern 1 and pattern 3, which we denoted as $X_{2,i(2)}$. Figure 2 illustrate how the above procedures work. When all the covariates are observed, $X_{k,i(r)}=X_{k,i}$. Similarly, we can define $\mu_{k,i(r)}(\beta )$ as the corresponding imputed conditional mean.

The intuition behind the proposed method stems from generalized estimating equations (GEE) and quadratic inference functions (QIF). Suppose $V_{k,i}$ is the unknown true covariance matrix of $Y_{k,i}$. Ref. [19] proposed that $V_{k,i}$ can be estimated by $A_{k,i}^{1/2}R_{k,i}(\alpha )A_{k,i}^{1/2}$, where $A_{k,i}$ is the diagonal matrix of the conditional variance of $Y_{k,i}$ and $R_{k,i}$ is a working correlation matrix that fully specified by a vector of parameter α. Ref. [20] proposed the QIF using the fact that the inverse of the correlation matrix $R_{k,i}^{-1}$ can be approximated by $\sum_{j=1}^{J}\;a_{k,j}M_{j}$, where $M_{1},\ldots ,M_{J}$ are some basis matrices. For example, if we assume the within-cluster correlation structure is exchangeable, $R_{k,i}^{-1}$ can be approximated by $a_{1}M_{1}+a_{2}M_{2}$, where $M_{1}$ is the identity matrix and $M_{2}$ is a matrix with elemtents in the diagonal to be 0 and elements in the off-diagonal to be 1. The estimation of inverse of correlation matrix using linear combination has been intensively studied by [21]. The advantage of this linear approximation is that the parameter α can be treated as a nuisance parameter, leading to some improvement in computational efficiency. Then, the estimating function for the subject i in the *k*th pattern with the *r*th imputation is defined as:

$${\tilde{g}}_{k,i(r)}(\beta )=\sum_{j=1}^{J}a_{k,j}{\left\{\frac{\partial \mu_{k,i}(\beta )}{\partial \beta_{O(k)}}\right\}}^{T}A_{k,i}^{-1/2}M_{j}A_{k,i}^{-1/2}\left\{Y_{k,i}-\mu_{k,i(r)}(\beta )\right\}.$$

Here, we only take derivative with respect to $\beta_{O(k)}$ to enhance numerical stability. Recall that $a_{k,j}$ is a linear coefficient that used to approximate the inverse of correlation matrix, and thus, it is the nuisance parameter. To avoid estimating these nuisance parameters, we define the extended score vector:

$$g_{k,i(r)}(\beta )=\left(\begin{array}{c} {\left\{\frac{\partial \mu_{k,i}(\beta )}{\partial \beta_{O(k)}}\right\}}^{T}A_{k,i}^{-1/2}M_{1}A_{k,i}^{-1/2}\left\{Y_{k,i}-\mu_{k,i(r)}(\beta )\right\}\\ \vdots \\ {\left\{\frac{\partial \mu_{k,i}(\beta )}{\partial \beta_{O(k)}}\right\}}^{T}A_{k,i}^{-1/2}M_{J}A_{k,i}^{-1/2}\left\{Y_{k,i}-\mu_{k,i(r)}(\beta )\right\} \end{array}\right).$$

Similarly, we obtain extended score vectors for all imputed covariate vectors and subjects. To integrate all score vectors, we aggregate the information by stacking them into a long vector:

$$g(\beta )=\left(\begin{array}{c} g_{1}(\beta )\\ \vdots \\ g_{K}(\beta ) \end{array}\right)=\left(\begin{array}{c} \frac{1}{n_{1}}\sum_{i=1}^{n_{1}}\;g_{1,i}(\beta )\\ \vdots \\ \frac{1}{n_{K}}\sum_{i=1}^{n_{K}}\;g_{K,i}(\beta ) \end{array}\right),$$

where $g_{k,i}(\beta )={\left(g_{k,i(1)}^{T}(\beta ),\ldots ,g_{k,i\left(R_{k}\right)}^{T}(\beta )\right)}^{T}$. Note that this might be an overdetermined system because the number of equations can exceed the number of parameters. To overcome this difficulty, we adopt generalized method of moment [22] and add a penalty term. Therefore, the objective function becomes:

(1) $$S(\beta )=g(\beta {)}^{T}C(\beta {)}^{-1}g(\beta )+\sum_{j=1}^{p}p_{\lambda_{n}}\left(\left|\beta_{j}\right|\right),$$

where:

$$C(\beta )=diag\left\{\frac{1}{n_{1}}\sum_{i=1}^{n_{1}}\;g_{1,i}(\beta )g_{1,i}^{T}(\beta ),\ldots ,\frac{1}{n_{K}}\sum_{i=1}^{n_{K}}\;g_{K,i}(\beta )g_{K,i}^{T}(\beta )\right\}$$

is a block-diagnoal matrix under the assumption of independence among samples from different missing patterns and $p_{\lambda_{n}}(\cdot )$ is an arbitrary, investigator’s chosen, penalty function with a tuning parameter λ. Among many optional penalty functions, we adopt the non-convex smoothly clipped absolute deviation (SCAD) penalty [23]:

$$p_{\lambda_{n}}(|\beta |)=\lambda |\beta |I(|\beta |\leq \lambda )+\frac{2a\lambda |\beta |-\beta^{2}-\lambda^{2}}{2(a-1)}I(\lambda <|\beta |\leq a\lambda )+\frac{\lambda^{2}(a+1)}{2}I(a\lambda <|\beta |)$$

for some a>2, which possess desirable oracle property.

## 3. Asymptotic Properties

In this section, we investigate the asymptotic properties of the proposed estimator. In Section 3.1, we assume the sample size n is increasing while the number of parameters p is fixed, and demonstrate that the proposed estimator is $\sqrt{n}$-consistent and asymptotically normal. As sample size goes to infinity, the proposed method is capable of selecting out the relevant variables with probability goes to 1. We also show that the proposed estimator is asymptotically more efficient than single imputation method via incorporating information of samples with missing values. In Section 3.2, we suppose both the sample size n and the number of parameters p are increasing but n increases faster than p. We show that the consistency and sparsity still hold with diverging p. Without loss of generality, we assume $\overset{ˆ}{\beta }$ can be partitioned into two parts, i.e., $\overset{ˆ}{\beta }={\left({\overset{ˆ}{\beta }}_{𝒜}^{T},{\overset{ˆ}{\beta }}_{𝒩}^{T}\right)}^{T}$, where ${\overset{ˆ}{\beta }}_{𝒜}$ corresponds to relevant variables with a non-zero true value, while ${\overset{ˆ}{\beta }}_{𝒩}$ consists of coefficients of irrelevant variables with a zero true value. For any function g(β), we use $\dot{g}(\beta )$ to denote the first derivative of g(⋅) evaluated at β. We use similar notation for its other order derivatives.

### 3.1. Fixed Number of Parameters

To establish the asymptotic properties of the proposed estimator in the setting of increasing sample sizes and fixed number of parameter, we require the following regularity conditions:

C.1 $E{\left[X_{k,j}\right]}^{4}<\infty$ and $E{\left[E\left[X_{k,j(r)}\right]\right]}^{4}<\infty$, for any 1≤k≤K, 1≤j≤p, and 1≤r, where the inner expectation is with respect to the imputed values.

C. 2 All the variance matrix $A_{k,i}\geq 0$ and $\left\|A_{k,i}\right\|<\infty$, for any 1≤k≤K and $1\leq i\leq n_{k}$.

C. 3 Let $\varepsilon_{k,i}=A_{k,i}^{-1/2}\left(Y_{k,i}-\mu_{k,i}\left(\beta_{0}\right)\right)$. For any 1≤k≤K and $1\leq i\leq n_{k}$, $E\left(\varepsilon_{k,i}\right)=0$ and the fourth moment of $\varepsilon_{k,i}$ exists.

C.4 $\left\|\mu_{k,i}\left(\beta_{0}\right)-\mu_{k,i(r)}\left(\beta_{0}\right)\right\|=o_{p}(n_{k}^{-1/2})$, for any 1≤k≤K and $1\leq i\leq n_{k}$.

C.5 The penalty function satisfied:

- ${lim\;inf}_{n\to \infty }\;{inf}_{\beta_{j}\to 0^{+}}\;p_{\lambda_{n}}^{\prime }\left(\beta_{j}\right)/\lambda_{n}>0$;
- ${max}_{j\in 𝒜}\;\{p_{\lambda_{n}}^{\prime }\left(\beta_{0j}\right)\}=o_{p}\left(n^{-1/2}\right)$;
- ${max}_{j\in 𝒜}\;\{p_{\lambda_{n}}^{\prime \prime }\left(\beta_{0j}\right)\}=o_{p}(1)$.

C.6 $\sqrt{n}g\left(\beta_{0}\right)\overset{d\;}{\to }N(0,\;\Sigma \Omega )$, where $\Sigma =diag\left\{\Sigma_{1},\ldots ,\Sigma_{K}\right\}$ and $\Omega =diag\left\{\Omega_{1},\ldots ,\Omega_{K}\right\}$, with $\Sigma_{k}=cov\left(g_{k,i}\left(\beta_{0}\right)\right)$ and $\Omega_{k}$ to be a diagonal matrix with $n_{k}$ dimension and each element equals to ${lim}_{n\to \infty }\;n/n_{k}$.

C.1–C.3 are conditions that require the existence of the moment, which are easily satisfied. C.4 requires the imputed conditional mean converges to the true conditional mean in probability, which is satisfied as long as the imputed model is correctly specified and the missing mechanism is either missing completely at random. C.5 is a standard condition for SCAD penalty which is commonly used in variable selection method (Gao et al. [24]; Cho and Qu [25]; Tian et al. [26]). More specifically, (a) ensures the property of sparsity is satisfied, (b) and (c) ensure the property of consistency is satisfied, and (c) also guarantees that the objective function (1) is dominated by the first term. C.6 is used to establish the asymptotic normality of the proposed estimator.

#### Theorem 1.

*Under C.1–C.5, there exists a local minimizer* $\overset{ˆ}{\beta }$ *of* S(β) *such that* $\left\|\overset{ˆ}{\beta }-\beta_{0}\right\|=O_{p}\left(n^{-1/2}\right)$.

Theorem 1 states the existence of a minimizer of the objective function and the minimizer will converge to the true coefficients at a rate of $\sqrt{n}$ as the sample size increases. Next, we demonstrate that this estimator possesses the sparsity property and the estimator for the non-zero coefficient is asymptotically normal, as outlined in the following theorem.

#### Theorem 2.

*Under C.1–C.5, if* $\lambda_{n}\to 0$ *and there exist a sequence such that* $\lambda_{n}\sqrt{n}/a_{n}\to \infty$ *as* n→∞, *where* $a_{n}=o_{p}(\sqrt{n})$, *then the proposed estimator* $\overset{ˆ}{\beta }={\left({\overset{ˆ}{\beta }}_{𝒜}^{T},{\overset{ˆ}{\beta }}_{𝒩}^{T}\right)}^{T}$ *satisfies the following properties:*

1. (*Sparsity*) $P({\overset{ˆ}{\beta }}_{𝒩}=0)\to 1$;
2. *(Asymptotic Normality) Let* $H=E\left[\partial g^{T}\left(\beta_{0}\right)/\partial \beta_{𝒜}\right]$ *and* $V={\left(H\Sigma^{-1}\Omega^{-1}H^{T}\right)}^{-1}$ *and if C. 6 holds, then* $\sqrt{n}({\overset{ˆ}{\beta }}_{𝒜}-\beta_{0𝒜})\overset{d\;}{\to }N(0,V)$.

The sparsity of the proposed estimator guarantees that the probability of selecting the true model approaches 1. We also obtained in Theorem 2 the asymptotic normality of ${\overset{ˆ}{\beta }}_{𝒜}$, the estimator of coefficients for the relevant variables, which allows us to estimate its variance if H and Σ are known. However, in practice, these are unknown to us. We can obtain the empirical variance covariance matrix of ${\overset{ˆ}{\beta }}_{𝒜}$ by replacing H with $\hat{H}(\overset{ˆ}{\beta })=\partial g^{T}(\overset{ˆ}{\beta })/\partial \beta_{𝒜}$ and replacing Σ with $C(\overset{ˆ}{\beta })$, i.e., $\hat{V}={\left(\hat{H}C^{-1}\Omega^{-1}{\hat{H}}^{T}\right)}^{-1}$. Next, we compare the empirical variance of the proposed estimator with the empirical variance of the single imputation approach.

#### Theorem 3.

If a single imputation is used based on complete cases and denotes the asymptotic covariance matrix of $\beta_{𝒜}$ as $\tilde{V}$, then under the conditions of Theorem 2 $\tilde{V}-V$ is positive semi-definite.

Theorem 3 claims that the proposed estimator is asymptotically more efficient than the single imputation approach, as it incorporates information from incomplete cases during imputation. The result of this Theorem is intuitive because the proposed method incorporates more samples into the imputation process.

### 3.2. Diverging Number of Parameters

In this subsection, we consider the setting where sample size n and number of coefficients p increase simultaneously. For certain properties to remain true, we require that n increases faster than p. We replace the notation p by $p_{n}$ to indicate that the number of parameters also increases. We make the following assumptions:

D.1 For any i,j,k,${\dot{Q}}_{k}\left(\beta_{0}\right)=o_{p}(p_{n}^{1/2}n^{-1/2})$ and:

$$\left\|\frac{\partial^{2}Q_{k}\left(\beta_{0}\right)}{\partial \beta_{i}\partial \beta_{j}}-E\{\frac{\partial^{2}Q_{k}\left(\beta_{0}\right)}{\partial \beta_{i}\partial \beta_{j}}\}\right\|=O_{p}(n^{-1/2}).$$

D.2 There exist an open ball of $\beta_{0}$ and there exist a constant M such that each entries of ${\dddot{Q}}_{k}(\beta )$ is bounded by M, for any β in this open ball.

D.3 The penalty function satisfied:

- ${lim\;inf}_{n\to \infty }\;{inf}_{\beta_{j}\to 0^{+}}\;p_{\lambda_{n}}^{\prime }\left(\beta_{j}\right)/\lambda_{n}>0$;
- ${max}_{j\in 𝒜}\;\{p_{\lambda_{n}}^{\prime }\left(\beta_{0j}\right)\}=o_{p}(p_{n}^{1/2}n^{-1/2})$;
- ${max}_{j\in 𝒜}\;\{p_{\lambda_{n}}^{\prime \prime }\left(\beta_{0j}\right)\}=o_{p}(p_{n}^{1/2})$.

D.1 and D.2 are analogous to C.1–C.4. D.3 is the modification of C.5 for diverging number of parameters.

#### Theorem 4.

*Under D.1–D.3, if* $p_{n}=o\left(n^{1/4}\right)$, *there exists a local minimizer* $\overset{ˆ}{\beta }$ *of* S(β) *such that* $\left\|\overset{ˆ}{\beta }-\beta_{0}\right\|=O_{p}(p_{n}^{1/2}n^{-1/2})$.

From the result of Theorem 4, we find that the consistency still holds for the proposed estimator, even with a diverging number of parameters. Not surprisingly, the convergence rate is no longer $\sqrt{n}$, but $\sqrt{n/p_{n}}$. We also require that $p_{n}$ does not increase faster than $n^{1/4}$ to ensure the model remains sparse. To be specific, the majority of the coefficients is zero.

#### Theorem 5.

*Under D.1–D.3, if* $p_{n}=o\left(n^{1/4}\right),\lambda_{n}\to 0$, and $\lambda_{n}\sqrt{n/p_{n}}\to \infty$ *as* n→∞, *then with probability tending to 1, the estimator* $\overset{ˆ}{\beta }={\left({\overset{ˆ}{\beta }}_{𝒜}^{T},{\overset{ˆ}{\beta }}_{𝒩}^{T}\right)}^{T}$ *satisfies* $P({\overset{ˆ}{\beta }}_{𝒩}=0)\to 1$.

Theorem 5 states the sparsity of the proposed estimator with a diverging number of parameters. This property guarantees that the proposed method can still select the true model with a probability approaching 1, even when the number of parameters is diverging.

## 4. Implementation

Since directly minimizing the objective function is difficult, we incorporate an iterative procedure inspired by the implementation in [27], where they combined the minorization–maximization algorithm [28] with the Newton–Raphson algorithm. Given the current estimate of $\beta^{\left(t\right)}$ and tuning parameter $\lambda_{n}$, the objective function S(β) can be locally approximated by (except a constant term):

(2) $$Q(\beta^{\left(t\right)})+\dot{Q}{\left(\beta^{\left(t\right)}\right)}^{T}(\beta -\beta^{\left(t\right)})+\frac{1}{2}{\left(\beta -\beta^{\left(t\right)}\right)}^{T}\ddot{Q}{\left(\beta^{\left(t\right)}\right)}^{T}(\beta -\beta^{\left(t\right)})+\frac{1}{2}\beta^{T}D_{\lambda_{n}}(\beta^{\left(t\right)})\beta ,$$

where:

$$D_{\lambda_{n}}(\beta^{\left(t\right)})=diag\left\{\frac{p_{\lambda_{n}}^{\prime }(\left|\beta_{1}^{\left(t\right)}\right|)}{\epsilon \;+\;|\beta_{1}^{\left(t\right)}|},\ldots ,\frac{p_{\lambda_{n}}^{\prime }(\left|\beta_{p}^{\left(t\right)}\right|)}{\epsilon \;+\;|\beta_{p}^{\left(t\right)}|}\right\}$$

and ϵ is a sufficiently small number (e.g., $\epsilon =10^{-6}$). Thus, the search for estimator minimizing the objective function is equivalent to find an estimator that minimize (2). Notice that both $\ddot{Q}(\beta^{\left(t\right)})$ and $\ddot{Q}(\beta^{\left(t\right)})$ are unknown. Fortunately, from Lemma S2 in Supplementary Materials, $\dot{Q}(\beta^{\left(t\right)})$ can be approximated by:

(3) $$M(\beta^{\left(t\right)})=2{\dot{g}}^{T}(\beta^{\left(t\right)})C{\left(\beta^{\left(t\right)}\right)}^{-1}g(\beta^{\left(t\right)})$$

and $\ddot{Q}(\beta^{\left(t\right)})$ can be approximated by:

(4) $$F(\beta^{\left(t\right)})=2{\dot{g}}^{T}(\beta^{\left(t\right)})C{\left(\beta^{\left(t\right)}\right)}^{-1}\dot{g}(\beta^{\left(t\right)}).$$

Plugging (3) and (4) into (2) and applying the Newton–Raphson algorithm, we obtain the following formula to update $\beta^{\left(t+1\right)}$:

$$\beta^{\left(t+1\right)}=\beta^{\left(t\right)}-{\left[F(\beta^{\left(t\right)})+D_{\lambda_{n}}(\beta^{\left(t\right)})\right]}^{-1}[M(\beta^{\left(t\right)})+D_{\lambda_{n}}(\beta^{\left(t\right)})\beta^{\left(t\right)}].$$

We repeat the above procedure until $\left\|\beta^{\left(t+1\right)}-\beta^{\left(t\right)}\right\|$ is smaller than a pre-specified threshold or reach a pre-specified maximum number of iteration.

It is known that the sampling covariance matrix C(β) may be singular in some cases [29]. To overcome the difficulty in computing the inverse of C(β), we adopt the Moore–Penrose generalized inverse, which exists and is unique for any matrix.

In the implementation of the proposed method, we select tuning parameter $\lambda_{n}$ with extended Bayesian Information Criterion (EBIC) criteria proposed by [30]:

$${EBIC}_{\gamma }=n\;log\;(RSS/n)+df_{\lambda_{n}}\{log(n)+2\gamma \;log(p)\},\;0\leq \gamma \leq 1,$$

where $df_{\lambda_{n}}$ is the number of parameters of the model with tuning parameter $\lambda_{n}$ and $RSS=\sum_{k=1}^{K}\;{RSS}_{k}$ is the residual sum of square of all the missing pattern with:

$${RSS}_{k}=\frac{1}{R_{k}}\sum_{r=1}^{R_{k}}\sum_{i=1}^{n_{k}}\sum_{j=1}^{m}{\left\{y_{k,ij}-\mu_{k,ij(r)}\right\}}^{2}.$$

## 5. Simulation

In this section, we implement a simulation study to compare the performance of the proposed method in variable selection against complete case analysis (CC) with SCAD penalty, single imputation (SI) with SCAD penalty, and the penalized generalized estimating Equation (PGEE). We use the same data structure as shown in Figure 1, where we have three missing patterns and three sources. The number of measurement is set to be three throughout this section. We replicate the simulation 100 times and use false positive rate (FPR) and false negative rate (FNR) to evaluate the performance of each method, which reflect the proportion of covariates that are irrelevant but falsely selected and the proportion of covariates that are relevant but fail to be selected, respectively. In the tuning parameter selection procedure, the parameter γ was set to 0.5 in EBIC. At the end of the iterative algorithm in Section 4, the estimated coefficient is considered as zero, if its absolute value is smaller than 0.01.

In the first setting, we simulate a dataset with a small proportion of complete cases, where $n_{1}=40$,$n_{2}=120$,$n_{3}=120$, and the missing rate is around 87%. The data with continuous outcome are generated from the model:

$$Y_{ij}=X_{ij}^{T}\beta +\varepsilon_{i},$$

where j=1,…,3, $X_{ij}={\left(x_{ij,1},\ldots ,x_{ij,30}\right)}^{T}$ is a vector consisting of 30 covariates, and $\beta =(1,2,0,\ldots ,0,1,2,0,\ldots ,0,1,2,0,\ldots ,0{)}^{T}$. Here, each source consists of 10 covariates with the first two covariates having non-zero coefficients. $x_{ij,1}$ is a time-fixed covariate and we generate it from the standard normal distribution, whereas other covariates are time-varying covariates and follow multivariate normal distribution with mean zero and exchangeable covariance matrix with marginal variance 1 and correlation coefficient 0.5. We generate random error $\varepsilon_{i}$ from the multivariate normal distribution with mean 0 and exchangeable covariance matrix with marginal variance 1 and correlation coefficient ρ. We always assume the true within-cluster correlation structure is known and considered ρ to be 0.3, 0.5, and 0.7 in each setting, which corresponded to mild, moderate, and strong within-cluster correlation. Let $\phi_{i}=1/\left(1+exp\left\{1+x_{i1,1}+\cdots +x_{i1,10}\right\}\right)$. Then, $n_{1}$,$n_{2}$, and $n_{3}$ samples were sequentially drawn with probability proportional to the $\phi_{i}$ and assigned to the pattern 1, pattern 2, and pattern 3, respectively. Obviously, subjects with higher covariates value from source 1 at the baseline are more likely to be assigned to pattern 1, followed by pattern 2 and then pattern 3. This data generating process implies a MAR mechanism for the missing covariates. The results of Table 1 summarize the performance of each method for three different ρ. All of these methods effectively control the FNR. However, FPR of the proposed method is lower than the other three methods. In other words, the proposed method is able to select most of relevant variables while controlling the error of selecting irrelevant variables. In addition, we notice that the proposed method is more capable of utilizing within-cluster correlation compared with PGEE since the proposed method performs better as the within-cluster correlation becomes stronger. This result demonstrates the superiority of the proposed method when the percentage of complete cases is small in the block-missing data.

In the second setting, we continue to investigate the proposed method’s performance with a continuous outcome, but we proportionally increase the sample size in each missing pattern to demonstrate the proposed method’s effectiveness in larger samples, where $n_{1}=120$, $n_{2}=300$, $n_{3}=300$. The results are described in Table 2. Unsurprisingly, the FPR and FNR of all the methods decreased compared with the first setting. We observe that the performance of the PGEE is very close to that of the single imputation method while the proposed method has a much lower FPR. In the meanwhile, complete cases analysis is still the worst option since the improvement is minor as the sample size increase, and even negligible when the within-cluster correlation is strong. Therefore, the proposed method is still able to maintain an appealing performance in the large sample size. The results from this setting further verify the efficiency gain of the proposed method in incorporating more information from the missing data compared to the single imputation.

In the third setting, we consider a correlated binary outcome with $n_{1}=120$, $n_{2}=300$, and $n_{3}=300$. The data are generated from the model:

$$log\frac{\pi_{ij}}{1-\pi_{ij}}=X_{ij}^{T}\beta +\varepsilon_{i},$$

where j=1,…,m,$X_{ij}={\left(x_{ij,1},\ldots ,x_{ij,15}\right)}^{T}$ is a vector consisting of 15 covariates, and $\beta =(1,0,\ldots ,0,-0.7,0,\ldots ,0,0.5,0,\ldots ,0{)}^{T}$. Here, each source consists of five covariates, with the first covariate in each having non-zero coefficients. $x_{ij,1}$ is a time-fixed covariate and we generate it from the standard normal distribution, whereas other covariates are time-varying covariates and follow multivariate normal distribution with mean zero and exchangeable covariance matrix with marginal variance 1 and correlation coefficient 0.5. We generate random error $\varepsilon_{i}$ from the multivariate normal distribution with mean 0 and exchangeable covariance matrix with marginal variance 1 and correlation coefficient 0.3. In this setting, $\phi_{i}=1/\left(1+exp\left\{1+x_{i1,11}+\cdots +x_{i1,15}\right\}\right)$. The results are summarized in Table 3. Although the PGEE outperforms other methods in terms of FPR, its performance in FNR is poor. In contrast, the proposed method possesses a better balance between FPR and FNR. We still observed a better performance of the proposed method.

## 6. Application

We apply our proposed method to the ADNI study. This study was launched in 2003 and has undertaken three different phases so far: ADNI 1, ADNI GO/2, and ADNI 3, which is designed to develop the effective treatment that can slow or stop the progression of AD. Our goal is to identify sensitive biomarkers of AD in the early stage from three data sources: magnetic resonance imaging (MRI), positron emission tomography (PET), and cerebrospinal fluid (CSF). We choose the mini-mental state examination (MMSE) [31] score as response variable, which has been widely used in the early diagnosis of AD [32]. The MRI data were analyzed by UCSF, who performed cortical reconstruction and volumetric segmentation with FreeSurfer. The processed MRI data primarly summarized average cortical thickness, standard deviation in cortical thickness, the volumes of cortical parcellations, the volumes of specific white matter parcellations, and the total surface area of the cortex [33]. The PET data were processed by UCB and quantities variables were obtained by standard uptake value ratio (SUVR) in amyloid florbetapir. The CSF data were acquired by ADNI Biomarker Core and Laboratory Medicine and Center for Neurodegenerative Diseases Research at UPENN. The block-wise missing emerged in this data. Less than half of patients lacked MRI measurements, few patients missed PET measurements, and only a small proportion of patients had CSF measurements. One of the reasons for the block-wise missing data is that obtaining CSF measurements requires more invasive procedures (such as lumbar puncture), which are refused by the majority of patients. The goal of this analysis is to identify biomarkers that are highly predictive of MMSE.

We only use the ADNI GO/2 dataset and consider measurements at baseline, month 24, and month 48, since the majority of patients have records at these time points. We also notice that there exist some low-quality data, such as those missed baseline measurement or belonged to a missing pattern with few patients. For simplicity of analysis, we discard these low-quality data, which leads us to a study cohort of 669 patients. Among them, 280 patients missed the measurement at month 24 and 487 patients missed the measurements at month 48. There are 340 features in MRI data, 229 features in PET data, and 3 features in CSF data. These three datasets and MMSE data are joined by a unique identifier “visit code” provided by the ADNI study. In total, we have three missing patterns. Table 4 describes the missing pattern of this dataset. The number of patients with fully observed variables is 63, with a missing rate around 90.6%. From this extremely high proportion of missing data, we will see how the proposed method can substantially improve the prediction ability by incorporating the information of related samples with missing values. To assess the predictive performance of the proposed method, data are randomly split into a test data with a sample size 30 (roughly 5%) and the remaining data as training data, where the test data are drawn from the data with fully observed variables (missing pattern 1). This random split process was replicated 30 times. A variable is marked as a potential predictor of AD if its absolute coefficient value is greater than 0.01.

Table 5 summarizes the average number of biomarkers selected by each method, along with the most frequently selected biomarkers. We also report the post-model-selection *p*-value. Our method successfully identifies biomarkers that align with findings reported in existing Alzheimer’s Disease research literature. In comparison to PGEE, the other three methods consistently select amyloid-*β* as a biomarker of AD, whose accumulation in cells is an early event of AD [34]. Phosphorylated tau, another widely accepted biomarker, has been validated by multiple large-scale, multi-center studies [35]. Studies found that neurons in AD patients are more likely to loss the superior temporal sulcus [36]. Two distinct normalization methods of summary measures for the standardized uptake value ratio (SUVR) of the florbetapir tracer, in the composite reference region and the whole cerebellum reference region, may potentially serving as AD biomarkers [37]. Besides these biomarkers, the proposed method additionally identifies several well-established and potential biomarkers. The size of the region of interest (ROI) in the left and right hemisphere precuneus area of the cortex, as well as cortical volume of left precuneus, summarize the health status of precuneus, which may be atrophy in the early stage of AD. The size and volume of the ROI in the left and right inferior lateral ventricle reflect disease progression (Bartos et al. [38]; Song et al. [39]). White matter changes in cerebral or subcortical areas can appear in other neurological conditions and normal aging, their connections with AD potentially make them useful biomarkers for distinguishing AD from normality, especially when considered along with other biomarkers in future investigations. While the surface area of the left caudal middle frontal and the cortical volume of the right caudal anterior cingulate are both associated with AD, more research is required to further explore these associations.

## 7. Discussion

It is well known that variable selection is a challenge for model robustness, estimator stableness and efficiency, as well as precise predictability. However, another non-negligible issue when integrating longitudinal studies is missingness in the covariate, especially in block-wise missing data. Specifically, with block-wise missing data, the percentage of complete observations is relatively small while traditional statistical methods heavily rely on information of complete cases. In this paper, we develop new methods to extend the MBI approach in a longitudinal study under the setting of block-wise missing data. Under certain regularity conditions, the desirable properties, consistency, sparsity, and asymptotic normality still hold. In addition, the proposed method demonstrates superior efficiency compared to the single imputation approach. It is worth noting that dropout missing data are also very common in longitudinal studies, which typically cause bias in many cases. In future work, it will be of great interest to develop methods to handle dropout missingness and incorporate inverse probability weighting in the proposed method.

One limitation of this paper is that we assume a homogeneous missing pattern across measurements within a single patient. Although this assumption may be restrictive in real data analysis, it is not hard to fulfill in multi-source data.

## Supplementary Material

supplement

## Figures and Tables

**Table 1.** Simulation scenario 1 with continuous outcomes: comparing the proposed method, complete cases analysis, single imputation method, and PGEE in terms of false positive rate (FPR), false negative rate (FNR), FPR + FNR, and computation time in seconds ($n_{1}=40$, $n_{2}=100$, $n_{3}=100$, $p_{1}=10$, $p_{2}=10$, $p_{3}=10$).

	Method	FPR	FNR	FPR + FNR	Time

ρ=0.3	Proposed	0.083	<0.001	0.083	2.38
	CC	0.204	<0.001	0.204	0.26
	SI	0.118	0.002	0.120	1.22
	PGEE	0.085	<0.001	0.085	0.62
ρ=0.5	Proposed	0.093	0.007	0.100	2.42
	CC	0.205	<0.001	0.205	0.27
	SI	0.146	<0.001	0.146	1.29
	PGEE	0.126	0.007	0.133	0.65
ρ=0.7	Proposed	0.110	<0.001	0.110	2.50
	CC	0.198	0.005	0.203	0.28
	SI	0.141	<0.001	0.141	1.33
	PGEE	0.132	0.017	0.149	0.67

**Table 2.** Simulation scenario 2 with continuous outcomes: comparing the proposed method, complete cases analysis, single imputation method, and PGEE in terms of false positive rate (FPR), false negative rate (FNR), FPR + FNR, and computation time in seconds ($n_{1}=120$, $n_{2}=300$, $n_{3}=300$, $p_{1}=10$, $p_{2}=10$, $p_{3}=10$).

	Method	FPR	FNR	FPR + FNR	Time

ρ=0.3	Proposed	0.003	<0.001	0.003	4.31
	CC	0.101	<0.001	0.101	0.58
	SI	0.018	<0.001	0.018	2.55
	PGEE	0.010	<0.001	0.010	1.55
ρ=0.5	Proposed	0.005	<0.001	0.005	4.37
	CC	0.135	<0.001	0.135	0.61
	SI	0.025	<0.001	0.025	2.55
	PGEE	0.023	<0.001	0.023	1.52
ρ=0.7	Proposed	0.015	<0.001	0.015	4.29
	CC	0.190	<0.001	0.190	0.54
	SI	0.049	<0.001	0.049	2.47
	PGEE	0.078	<0.001	0.078	1.37

**Table 3.** Simulation scenario 3 with binary outcomes: comparison of the proposed method, complete cases analysis, single imputation method, and PGEE in terms of false positive rate (FPR), false negative rate (FNR), FPR + FNR, and computation time in seconds ($n_{1}=120$, $n_{2}=300$, $n_{3}=300$, $p_{1}=5$, $p_{2}=5$, $p_{3}=5$, ρ=0.3).

Method	FPR	FNR	FPR + FNR	Time

Proposed	0.298	0.063	0.361	3.55
CC	0.334	0.218	0.552	0.32
SI	0.289	0.088	0.377	1.91
PGEE	0.071	0.537	0.608	0.74

**Table 4.** Data composition and missing pattern for the subset of ADNI data; “O” denotes the observed data and “-” denotes the missing data.

Missing Pattern	MRI (340)	PET (229)	CSF (3)	Number of Patients

1	O	O	O	63
2	O	O	-	384
3	-	O	-	222

**Table 5.** Comparision of the mean of the number of selected biomarkers (MNSB) whose absolute value of coefficient is greater than 0.01 based on 30 replications in application to ADNI data. Time is the computation time in seconds.

Method	MNSB	Top Selected Biomarkers	Time
Proposed	16	ABETA, PTAU, ST30SV *, ST15SA ST89SV, ST151SV, ST52CV *, ST73CV, SUMMARYSUVR COMPOSITE REFNORM *, SUMMARYSUVR WHOLECEREBNORM *, CTX LH PRECUNEUS VOLUME, CTX RH PRECUNEUS SUVR, LEFT INF LAT VENT VOLUME, RIGHT INF LAT VENT VOLUME, CTX LH SUPERIORTEMPORAL SUVR *, LEFT CEREBRAL WHITE MATTER VOLUME	1550
CC	3	ABETA, TAU *, SUMMARYSUVR COMPOSITE REFNORM *	280
SI	9	ABETA *, TAU *, PTAU *, ST1SV, ST4SV *, ST52CV SUMMARYSUVR COMPOSITE REFNORM, SUMMARYSUVR WHOLECEREBNORM CC MID ANTERIOR VOLUME	1216
PGEE	1	ST52TA *	18

* Post-model-selection *p*-value < 0.05.

## Data Availability

Data are publicly available (https://adni.loni.usc.edu, accessed on 29 February 2024).
