# Supplementary material for: Imputation-Based Variable Selection Method for Block-Wise Missing Data When Integrating Multiple Longitudinal Studies
Source: Mathematics (Basel). Author manuscript; Available in PMC 2025 Feb 7. (PMC11804884; doi:10.3390/math12070951)
Supplement: supplement [file NIHMS2039846-supplement-supplement.pdf]

Supplementary Materials

Lemma S1. *Under C.1-C.4,* $\|\mathbf{g}_k(\boldsymbol{\beta})\| = O_p(n_k^{-1/2})$ *and* $\|\dot{\mathbf{g}}_k(\boldsymbol{\beta}_0)\| = O_p(1)$.

Proof. Without loss of generality, independent correlation structure and canonical link is considered. The true correlation matrix is approximated by $\boldsymbol{A}^{-1/2}\boldsymbol{M}_1\boldsymbol{A}^{-1/2}$. Under canonical link, if we assume the scale parameter equals 1, then $\partial\boldsymbol{\mu}_{k,i}(\boldsymbol{\beta})/\partial\boldsymbol{\beta} = \boldsymbol{A}_{k,i}\boldsymbol{X}_{k,i}$. Let $\tilde{\boldsymbol{X}}_{k,i} = \boldsymbol{X}^O_{k,i(r)}$ for all r, the submatrix of $\boldsymbol{X}_{k,i(r)}$ with columns consist of observed covariates.

By C.4,

$$\begin{aligned}\frac{1}{n_k}\sum_{i=1}^{n_k}\mathbf{g}_{k,i(r)}(\boldsymbol{\beta}_0) &= \frac{1}{n_k}\sum_{i=1}^{n_k}\tilde{\boldsymbol{X}}_{k,i}^T\boldsymbol{A}_{k,i}^{1/2}\boldsymbol{M}_1\boldsymbol{A}_{k,i}^{-1/2}(\boldsymbol{Y}_{k,i}-\boldsymbol{\mu}_{k,i(r)}(\boldsymbol{\beta}_0))\\ &= \frac{1}{n_k}\sum_{i=1}^{n_k}\tilde{\boldsymbol{X}}_{k,i}^T\boldsymbol{A}_{k,i}^{1/2}\boldsymbol{M}_1\boldsymbol{\varepsilon}_{k,i} + \frac{1}{n_k}\sum_{i=1}^{n_k}\tilde{\boldsymbol{X}}_{k,i(r)}^T\boldsymbol{A}_{k,i}^{1/2}\boldsymbol{M}_1\boldsymbol{A}_{k,i}^{-1/2}(\boldsymbol{\mu}_{k,i}(\boldsymbol{\beta}_0)-\boldsymbol{\mu}_{k,i(r)}(\boldsymbol{\beta}_0))\\ &= \frac{1}{n_k}\sum_{i=1}^{n_k}\tilde{\boldsymbol{X}}_{k,i}^T\boldsymbol{A}_{k,i}^{1/2}\boldsymbol{M}_1\boldsymbol{\varepsilon}_{k,i} + o_p(n_k^{-1/2}).\end{aligned}$$

By Chebyshev's inequality and C.1-C.3, $\|1/n_k\sum_{i=1}^{n_k}\tilde{\boldsymbol{X}}_{k,i}^T\boldsymbol{A}_{k,i}^{1/2}\boldsymbol{M}_1\boldsymbol{\varepsilon}_{k,i}\| = O_p(n_k^{-1/2})$. Also, K and number of imputations are both finite, implying $n_k = O(n)$. Therefore, $\|\mathbf{g}_k(\boldsymbol{\beta})\| = O_p(n^{-1/2})$.

Because each element of $\dot{\mathbf{g}}_k(\boldsymbol{\beta}_0)$,

$$\frac{1}{n_k}\sum_{i=1}^{n_k}\dot{\mathbf{g}}_{k,i(r)}(\boldsymbol{\beta}_0) = \frac{1}{n_k}\sum_{i=1}^{n_k}\boldsymbol{X}_{k,i(r)}^T\boldsymbol{A}_{k,i}^{1/2}\boldsymbol{M}_1\boldsymbol{A}_{k,i}^{-1/2}\tilde{\boldsymbol{X}}_{k,i}$$

is bounded in probability by Law of Large Number, C.1-C.2, and the finite number of imputations. It follows that $\|\dot{\mathbf{g}}_k(\boldsymbol{\beta}_0)\| = O_p(1)$. □

Lemma S2. *Let* $Q_k(\boldsymbol{\beta}) = \mathbf{g}_k^T(\boldsymbol{\beta})^T\boldsymbol{C}_k(\boldsymbol{\beta})^{-1}\mathbf{g}_k(\boldsymbol{\beta})$. *Under C.1-C.4, we have*

$$\dot{Q}_k(\boldsymbol{\beta}_0) = 2\dot{\mathbf{g}}_k^T(\boldsymbol{\beta}_0)\boldsymbol{C}_k(\boldsymbol{\beta}_0)^{-1}\mathbf{g}_k(\boldsymbol{\beta}_0) + o_p(1)$$

and

$$\ddot{Q}_k(\boldsymbol{\beta}_0) = 2\dot{\mathbf{g}}_k^T(\boldsymbol{\beta}_0)\boldsymbol{C}_k(\boldsymbol{\beta}_0)^{-1}\dot{\mathbf{g}}_k(\boldsymbol{\beta}_0) + o_p(1).$$

Proof. By C.3 and Law of Large Number, we have $\|\boldsymbol{C}_k(\boldsymbol{\beta}_0)\| = O_p(1)$. Thus,

$$\begin{aligned}\dot{Q}_k(\boldsymbol{\beta}_0) &= 2\dot{\mathbf{g}}_k^T(\boldsymbol{\beta}_0)\boldsymbol{C}_k(\boldsymbol{\beta}_0)^{-1}\mathbf{g}_k(\boldsymbol{\beta}_0) + \mathbf{g}_k^T(\boldsymbol{\beta}_0)\dot{\boldsymbol{C}}_k(\boldsymbol{\beta}_0)^{-1}\mathbf{g}_k(\boldsymbol{\beta}_0)\\ &= 2\dot{\mathbf{g}}_k^T(\boldsymbol{\beta}_0)\boldsymbol{C}_k(\boldsymbol{\beta}_0)^{-1}\mathbf{g}_k(\boldsymbol{\beta}_0) + o_p(1),\end{aligned}$$

where $\dot{\boldsymbol{C}}_k(\boldsymbol{\beta}_0)^{-1}$ is a three-dimensional array and is bounded in probability. Similarly, we can show that

$$\begin{aligned}\ddot{Q}_k(\boldsymbol{\beta}_0) &= 2\dot{\mathbf{g}}_k^T(\boldsymbol{\beta}_0)\boldsymbol{C}_k(\boldsymbol{\beta}_0)^{-1}\dot{\mathbf{g}}_k(\boldsymbol{\beta}_0) + 4\dot{\mathbf{g}}_k^T(\boldsymbol{\beta}_0)\frac{\partial\boldsymbol{C}_k(\boldsymbol{\beta}_0)^{-1}}{\partial\boldsymbol{\beta}}\mathbf{g}_k(\boldsymbol{\beta}_0)\\ &\quad + 2\ddot{\mathbf{g}}_k^T(\boldsymbol{\beta}_0)\boldsymbol{C}_k(\boldsymbol{\beta}_0)^{-1}\mathbf{g}_k(\boldsymbol{\beta}_0) + \mathbf{g}_k^T(\boldsymbol{\beta}_0)\ddot{\boldsymbol{C}}_k(\boldsymbol{\beta}_0)^{-1}\mathbf{g}_k(\boldsymbol{\beta}_0)\\ &= 2\dot{\mathbf{g}}_k^T(\boldsymbol{\beta}_0)\boldsymbol{C}_k(\boldsymbol{\beta}_0)^{-1}\dot{\mathbf{g}}_k(\boldsymbol{\beta}_0) + o_p(1).\end{aligned}$$

□

Proof of Theorem S1

Proof. It suffices to prove that for any $\epsilon > 0$, with probability at least $1 - \epsilon$, there exist a constant C_ϵ such that a local minimizer exists within the ball

$$\{\boldsymbol{\beta}_0 + n^{-1/2}\boldsymbol{u} : \|\boldsymbol{u}\| < C_\epsilon\}.$$

Let $Q_k = \mathbf{g}_k(\boldsymbol{\beta})^T \boldsymbol{C}_k(\boldsymbol{\beta})^{-1}\mathbf{g}_k(\boldsymbol{\beta})$, where $\boldsymbol{C}_k(\boldsymbol{\beta}) = 1/n_k \sum_{i=1}^{n_k} \mathbf{g}_{k,i}(\boldsymbol{\beta})\mathbf{g}_{k,i}^T(\boldsymbol{\beta})$. We notice that $Q(\boldsymbol{\beta}) = \sum_{k=1}^K Q_k(\boldsymbol{\beta})$ since $\boldsymbol{C}$ is a block-diagonal matrix.

By Taylor expansion,

$$\begin{aligned}
S(\boldsymbol{\beta}_0 + n^{-1/2}\boldsymbol{u}) - S(\boldsymbol{\beta}_0) &= Q(\boldsymbol{\beta}_0 + n^{-1/2}\boldsymbol{u}) - Q(\boldsymbol{\beta}_0) + \sum_{j=1}^p \left[p_{\lambda_n}(|\beta_{0j} + n^{-1/2}u_j|) - p_{\lambda_n}(|\beta_{0j}|)\right] \\
&= \sum_{k=1}^K \left[Q_k(\boldsymbol{\beta}_0 + n^{-1/2}\boldsymbol{u}) - Q_k(\boldsymbol{\beta}_0)\right] + \sum_{j=1}^p \left[p_{\lambda_n}(|\beta_{0j} + n^{-1/2}u_j|) - p_{\lambda_n}(|\beta_{0j}|)\right] \\
&\geq \sum_{k=1}^K \left[Q_k(\boldsymbol{\beta}_0 + n^{-1/2}\boldsymbol{u}) - Q_k(\boldsymbol{\beta}_0)\right] + \sum_{j\in\mathcal{A}} \left[p_{\lambda_n}(|\beta_{0j} + n^{-1/2}u_j|) - p_{\lambda_n}(|\beta_{0j}|)\right] \\
&= I_1 + I_2.
\end{aligned}$$

We further expand I_1 by Taylor expansion

$$\begin{aligned}
I_1 &= \sum_{k=1}^K \left[Q_k(\boldsymbol{\beta}_0 + n^{-1/2}\boldsymbol{u}) - Q_k(\boldsymbol{\beta}_0)\right] \\
&= \sum_{k=1}^K n^{-1/2}\boldsymbol{u}^T \dot{Q}_k(\boldsymbol{\beta}_0) + \sum_{k=1}^K \frac{1}{2} n^{-1}\boldsymbol{u}^T \ddot{Q}_k(\boldsymbol{\beta}_0)\boldsymbol{u} + \sum_{k=1}^K \frac{1}{6} n^{-\frac{3}{2}} \frac{\partial}{\partial \boldsymbol{\beta}} \left\{\boldsymbol{u}^T \ddot{Q}_k(\boldsymbol{\beta}^*)\boldsymbol{u}\right\} \boldsymbol{u} \\
&= I_{11} + I_{12} + o_p(n^{-1}),
\end{aligned}$$

where $\boldsymbol{\beta}^*$ is an intermediate value between $\hat{\boldsymbol{\beta}}$ and $\boldsymbol{\beta}_0$.

By Lemmas S1 and S2,

$$\|I_{11}\| \leq \sum_{k=1}^K n^{-1/2} \left\|2\boldsymbol{u}^T \dot{\mathbf{g}}_k^T(\boldsymbol{\beta}_0)\boldsymbol{C}_k(\boldsymbol{\beta}_0)^{-1}\mathbf{g}_k(\boldsymbol{\beta}_0) + o_p(1)\right\| = O_p(n^{-1})\|\boldsymbol{u}\|$$

and

$$\|I_{12}\| \leq \frac{1}{2}\sum_{k=1}^K n^{-1} \left\|2\boldsymbol{u}^T \dot{\mathbf{g}}_k^T(\boldsymbol{\beta}_0)\boldsymbol{C}_k(\boldsymbol{\beta}_0)^{-1}\dot{\mathbf{g}}_k(\boldsymbol{\beta}_0)\boldsymbol{u} + o_p(1)\right\| = O_p(n^{-1})\|\boldsymbol{u}\|^2.$$

By C.6,

$$\begin{aligned}
I_2 &= \sum_{j\in\mathcal{A}} \left[p_{\lambda_n}(|\beta_{0j} + n^{-1/2}u_j|) - p_{\lambda_n}(|\beta_{0j}|)\right] \\
&= \sum_{j\in\mathcal{A}} n^{-1/2}u_j p'_{\lambda_n}(|\beta_{0j}|)\mathrm{sign}(|\beta_{0j}|) + \sum_{j\in\mathcal{A}} \frac{1}{2} n^{-1}u_j^2 p''_{\lambda_n}(|\beta_{0j}|) + o_p(n^{-1}) \\
&= I_{21} + I_{22}.
\end{aligned}$$

Notice that

$$|I_{21}| \leq \sum_{j\in\mathcal{A}} \left| n^{-1/2} u_j p'_{\lambda_n}(|\beta_{0j}|)\mathrm{sign}(\beta_{0j}) \right| \leq \max_{j\in\mathcal{A}}\{p'_{\lambda_n}(\beta_{0j})\} \sum_{j\in\mathcal{A}} |n^{-1/2}u_j| \leq o_p(n^{-1})\|\boldsymbol{u}\|$$

and

$$I_{22} \leq \max_{j\in\mathcal{A}}\{p''_{\lambda_n}(|\beta_{0j}|)\}\frac{1}{2}n^{-1}\|\boldsymbol{u}\|^2 = o_p(n^{-1})\|\boldsymbol{u}\|^2.$$

By choosing sufficient large $\|\boldsymbol{u}\|$, $I_1 + I_2$ will be dominated by I_{12}, which is positive. Thus, $S(\boldsymbol{\beta}_0 + n^{-1/2}\boldsymbol{u}) - S(\boldsymbol{\beta}_0) > 0$.

□

Proof of Theorem S2

Proof. To prove sparsity, it suffices to prove that there exist $M_0 > 0$ such that for any $j \in \mathcal{N}$,

$$\frac{\partial S(\hat{\boldsymbol{\beta}})}{\partial \beta_j} > 0, \text{when } 0 < \hat{\beta}_j < M_0 n^{-1/2}$$
$$\frac{\partial S(\hat{\boldsymbol{\beta}})}{\partial \beta_j} < 0, \text{when } - M_0 n^{-1/2} < \hat{\beta}_j < 0.$$

By Taylor expansion,

$$\begin{aligned}
\frac{\partial S(\hat{\boldsymbol{\beta}})}{\partial \beta_j} &= \frac{\partial Q(\hat{\boldsymbol{\beta}})}{\partial \beta_j} + p'_{\lambda_n}(|\hat{\beta}_j|)\mathrm{sign}(\hat{\beta}_j) \\
&= \frac{\partial Q(\boldsymbol{\beta}_0)}{\partial \beta_j} + \sum_{l=1}^{p} \frac{\partial^2 Q(\boldsymbol{\beta}_0)}{\partial \beta_j \partial \beta_l}(\hat{\beta}_l - \beta_{0l}) \\
&+ \sum_{l,k=1}^{p} \frac{\partial^3 Q(\boldsymbol{\beta}^*)}{\partial \beta_j \partial \beta_l \partial \beta_k}(\hat{\beta}_l - \beta_{0l})(\hat{\beta}_k - \beta_{0k}) + p'_{\lambda_n}(|\hat{\beta}_j|)\mathrm{sign}(\hat{\beta}_j) \\
&= I_1 + I_2 + I_3 + I_4,
\end{aligned}$$

where $\boldsymbol{\beta}^*$ is an intermediate value between $\hat{\boldsymbol{\beta}}$ and $\boldsymbol{\beta}_0$.

By Lemma S2, $\|I_1\| = O_p(n^{-1/2})$. By the Cauchy-Schwarz inequality and Therorem 1, we have

$$\|I_2\|^2 \leq \|\ddot{Q}(\boldsymbol{\beta}_0)\|^2\|\hat{\boldsymbol{\beta}} - \boldsymbol{\beta}_0\|^2 = O_p(n^{-1})$$

and $\|I_3\| = o_p(n^{-1/2})$.

Because $\lambda_n \to 0$ and $\lambda_n \sqrt{n}/a_n \to \infty$,

$$I_4 = \lambda_n\{p'_{\lambda_n}(|\hat{\beta}_j|)/\lambda_n \mathrm{sign}(\hat{\beta}_j)\}$$

dominates I_1, I_2, and I_3. Therefore, $\partial S(\hat{\boldsymbol{\beta}})/\partial \beta_j$ has the same sign as $\hat{\beta}_j$. This finishes the proof of sparsity.

Next we prove the asymptotic normality. By the Taylor expansion,

$$\frac{\partial S(\hat{\boldsymbol{\beta}})}{\partial \boldsymbol{\beta}_{\mathcal{A}}} = \frac{\partial Q(\boldsymbol{\beta}_0)}{\partial \boldsymbol{\beta}_{\mathcal{A}}} + \frac{\partial^2 Q(\boldsymbol{\beta}_0)}{\partial \boldsymbol{\beta}_{\mathcal{A}} \partial \boldsymbol{\beta}_{\mathcal{A}}^T}(\hat{\boldsymbol{\beta}}_{\mathcal{A}} - \boldsymbol{\beta}_{0\mathcal{A}}) + \mathcal{P}'_{\lambda_n}(|\boldsymbol{\beta}_{0\mathcal{A}}|)\mathrm{sign}(\boldsymbol{\beta}_{0\mathcal{A}}) + \mathcal{P}''_{\lambda_n}(|\boldsymbol{\beta}_{0\mathcal{A}}|)(\hat{\boldsymbol{\beta}}_{\mathcal{A}} - \boldsymbol{\beta}_{0\mathcal{A}}) + \boldsymbol{r}_n,$$

where $\mathcal{P}'_{\lambda_n}(|\boldsymbol{\beta}|)$ is a vector consisting of $p'_{\lambda_n}(|\boldsymbol{\beta}|)$ and $\mathcal{P}''_{\lambda_n}(|\boldsymbol{\beta}|)$ is a diagonal matrix consisting of $p''_{\lambda_n}(|\boldsymbol{\beta}|)$. Because $\hat{\boldsymbol{\beta}}$ is a local minimizer, indicating that $\partial S(\hat{\boldsymbol{\beta}})/\partial \boldsymbol{\beta}_{\mathcal{A}} = 0$, we obtain

$$\sqrt{n}(\hat{\boldsymbol{\beta}}_{\mathcal{A}} - \boldsymbol{\beta}_{0\mathcal{A}}) = -\sqrt{n}\left[\frac{\partial^2 Q(\boldsymbol{\beta}_0)}{\partial \boldsymbol{\beta}_{\mathcal{A}} \partial \boldsymbol{\beta}_{\mathcal{A}}^T} + \mathcal{P}''_{\lambda_n}(|\boldsymbol{\beta}_{0\mathcal{A}}|)\right]^{-1}\left[\frac{\partial Q(\boldsymbol{\beta}_0)}{\partial \boldsymbol{\beta}_{\mathcal{A}}} + \mathcal{P}'_{\lambda_n}(|\boldsymbol{\beta}_{0\mathcal{A}}|)\text{sign}(\boldsymbol{\beta}_{0\mathcal{A}}) + \boldsymbol{r}_n\right].$$

By the definition of SCAD penalty, $\mathcal{P}''_{\lambda_n}(|\boldsymbol{\beta}_{0\mathcal{A}}|) \xrightarrow{p} \mathbf{0}$, $\sqrt{n}\mathcal{P}'_{\lambda_n}(|\boldsymbol{\beta}_{0\mathcal{A}}|) \xrightarrow{p} \mathbf{0}$, and $\sqrt{n}\boldsymbol{r}_n \xrightarrow{p} \mathbf{0}$. By Lemma S2,

$$\sqrt{n}\frac{\partial Q(\boldsymbol{\beta}_0)}{\partial \boldsymbol{\beta}_{\mathcal{A}}} = 2\sqrt{n}\frac{\partial \mathbf{g}(\boldsymbol{\beta}_0)}{\partial \boldsymbol{\beta}_{\mathcal{A}}}\boldsymbol{C}(\boldsymbol{\beta}_0)^{-1}\mathbf{g}(\boldsymbol{\beta}_0) + o_p(1).$$

By C.6 and Law of Large Numbers, $\boldsymbol{C}(\boldsymbol{\beta}_0) \xrightarrow{p} \boldsymbol{\Sigma}$, $\partial \mathbf{g}(\boldsymbol{\beta}_0)/\partial \boldsymbol{\beta}_{\mathcal{A}} \xrightarrow{p} \boldsymbol{H}$, and $\sqrt{n}\mathbf{g}(\boldsymbol{\beta}_0) \xrightarrow{d} N(\mathbf{0}, \boldsymbol{\Sigma\Omega})$. By C.6, Slutsky's Theorem, and continuous mapping, we have

$$\sqrt{n}\left[\frac{\partial Q(\boldsymbol{\beta}_0)}{\partial \boldsymbol{\beta}_{\mathcal{A}}} + \mathcal{P}'_{\lambda_n}(|\boldsymbol{\beta}_{0\mathcal{A}}|)\text{sign}(\boldsymbol{\beta}_{0\mathcal{A}}) + \boldsymbol{r}_n\right] \xrightarrow{d} N(\mathbf{0}, 4\boldsymbol{H\Sigma}^{-1}\boldsymbol{\Omega H}^T).$$

Similarly, we can show that

$$\frac{\partial^2 Q(\boldsymbol{\beta}_0)}{\partial \boldsymbol{\beta}_{\mathcal{A}} \partial \boldsymbol{\beta}_{\mathcal{A}}^T} + \mathcal{P}''_{\lambda_n}(|\boldsymbol{\beta}_{0\mathcal{A}}|) \xrightarrow{p} 2\boldsymbol{H\Sigma}^{-1}\boldsymbol{H}^T.$$

Again, by Slutsky's Theorem, $\sqrt{n}(\hat{\boldsymbol{\beta}}_{\mathcal{A}} - \boldsymbol{\beta}_{0\mathcal{A}}) \xrightarrow{d} N(\mathbf{0}, \boldsymbol{V})$, where $\boldsymbol{V} = (\boldsymbol{H\Sigma}^{-1}\boldsymbol{\Omega}^{-1}\boldsymbol{H}^T)^{-1}$. □

Proof of Theorem S3

Proof. We use the similar notation as Theorem S2 with $\widetilde{\boldsymbol{V}} = (\widetilde{\boldsymbol{H}}\widetilde{\boldsymbol{C}}^{-1}\boldsymbol{\Omega}^{-1}\widetilde{\boldsymbol{H}}^T)^{-1}$. It suffices to prove that $\boldsymbol{HC}^{-1}\boldsymbol{H}^T \geq \boldsymbol{HC}^{-1}\boldsymbol{H}^T$. Because $\widehat{\boldsymbol{V}}$ and $\widetilde{\boldsymbol{V}}$ are block-diagonal matrices, we have

$$\boldsymbol{HC}^{-1}\boldsymbol{H}^T = \sum_{k=1}^{K} \boldsymbol{H}_k\boldsymbol{C}_k^{-1}\boldsymbol{H}_k^T$$

and

$$\widetilde{\boldsymbol{H}}\widetilde{\boldsymbol{C}}^{-1}\widetilde{\boldsymbol{H}}^T = \sum_{k=1}^{K} \widetilde{\boldsymbol{H}}_k\widetilde{\boldsymbol{C}}_k^{-1}\widetilde{\boldsymbol{H}}_k^T.$$

Obviously, $\boldsymbol{HC}^{-1}\boldsymbol{H}^T \geq \widetilde{\boldsymbol{H}}\widetilde{\boldsymbol{C}}^{-1}\widetilde{\boldsymbol{H}}^T$ will hold if, for any k, $\boldsymbol{H}_k\boldsymbol{C}_k^{-1}\boldsymbol{H}_k^T \geq \widetilde{\boldsymbol{H}}_k\widetilde{\boldsymbol{C}}_k^{-1}\widetilde{\boldsymbol{H}}_k^T$.

Let $\boldsymbol{G}_k(\boldsymbol{\beta}) = (\mathbf{g}_{k,1}(\boldsymbol{\beta}), \ldots, \mathbf{g}_{k,n_k}(\boldsymbol{\beta}))$. Then, we reordering the row of $\boldsymbol{G}_k(\boldsymbol{\beta})$ by letting $\boldsymbol{B}_k$ be a matrix such that $\boldsymbol{B}_k\boldsymbol{G}_k(\boldsymbol{\beta}) = (\boldsymbol{G}_{k1}^T(\boldsymbol{\beta}), \boldsymbol{G}_{k2}^T(\boldsymbol{\beta}))^T$, where $\boldsymbol{G}_{k1}(\boldsymbol{\beta})$ are the estimating equations constructed based on complete cases and $\boldsymbol{G}_{k2}(\boldsymbol{\beta})$ are the remaining estimating equations. We make the following transformation

$$\boldsymbol{G}_{k20}(\boldsymbol{\beta}) = \boldsymbol{G}_{k2}(\boldsymbol{\beta}) - \left[\frac{1}{n_k}\boldsymbol{G}_{k1}(\boldsymbol{\beta})\boldsymbol{G}_{k2}^T(\boldsymbol{\beta})\right]\left[\frac{1}{n_k}\boldsymbol{G}_{k1}(\boldsymbol{\beta})\boldsymbol{G}_{k1}^T(\boldsymbol{\beta})\right]^{-1}\boldsymbol{G}_{k1}(\boldsymbol{\beta}).$$

It follows that $\boldsymbol{G}_{k1}(\boldsymbol{\beta})\boldsymbol{G}_{k20}^T(\boldsymbol{\beta}) = \mathbf{0}$. Let $\boldsymbol{U}_k$ be a matrix such that $\boldsymbol{U}_k\boldsymbol{G}_k(\boldsymbol{\beta}) = (\boldsymbol{G}_{k1}^T(\boldsymbol{\beta}), \boldsymbol{G}_{k20}^T(\boldsymbol{\beta}))^T$. Define $\boldsymbol{H}_k\boldsymbol{U}_k^T = (\widetilde{\boldsymbol{H}}_k, \widetilde{\boldsymbol{H}}_{k2})$. Thus

$$\begin{aligned}\boldsymbol{H}_k\boldsymbol{C}_{0k}^{-1}\boldsymbol{H}_k^T &= \boldsymbol{H}_k\boldsymbol{U}_k^T\left[\boldsymbol{U}_k\boldsymbol{C}_k\boldsymbol{U}_k^T\right]^{-1}\boldsymbol{U}_k\boldsymbol{H}_k^T\\ &= \widetilde{\boldsymbol{H}}_k\widetilde{\boldsymbol{C}}_k^{-1}\widetilde{\boldsymbol{H}}_k^T + \widetilde{\boldsymbol{H}}_{k2}\widetilde{\boldsymbol{C}}_{k2}^{-1}\widetilde{\boldsymbol{H}}_{k2}^T,\end{aligned}$$

where $\widetilde{\boldsymbol{C}}_{k2} = 1/n_k\boldsymbol{G}_{k20}(\boldsymbol{\beta})\boldsymbol{G}_{k20}^T(\boldsymbol{\beta})$. The last equality is because

$$\boldsymbol{U}_k\boldsymbol{C}_k\boldsymbol{U}_k^T = \boldsymbol{U}_k\left[\frac{1}{n_k}\boldsymbol{G}_k(\boldsymbol{\beta})\boldsymbol{G}_k^T(\boldsymbol{\beta})\right]\boldsymbol{U}_k^T.$$

Note that $\widetilde{\boldsymbol{H}}_{k2}\widetilde{\boldsymbol{C}}_{k2}^{-1}\widetilde{\boldsymbol{H}}_{k2}^T$ is a positive semidefinite matrix. Thus, $\boldsymbol{H}_k\boldsymbol{C}_k^{-1}\boldsymbol{H}_k^T \geq \widetilde{\boldsymbol{H}}_k\widetilde{\boldsymbol{C}}_k^{-1}\widetilde{\boldsymbol{H}}_k^T$. □

Lemma S3. *Under D.1,* $\|\ddot{Q}_k(\boldsymbol{\beta}_0) - E\{\ddot{Q}_k(\boldsymbol{\beta}_0)\}\| = O_p(p_n^3 n^{-1})$.

Proof. By D.1 and Chebyshev's inequality, for any $\varepsilon > 0$,

$$\begin{aligned}P\left(\left\|\ddot{Q}_k(\boldsymbol{\beta}_0) - E\{\ddot{Q}_k(\boldsymbol{\beta}_0)\}\right\| \geq \frac{\varepsilon}{p_n}\right) &\leq \frac{p_n^2}{\varepsilon^2}E\left(\left\|\ddot{Q}_k(\boldsymbol{\beta}_0) - E\{\ddot{Q}_k(\boldsymbol{\beta}_0)\}\right\|^2\right)\\ &= \frac{p_n^2}{\varepsilon^2}E\left(\sum_{i=1}^{p_n}\sum_{j=1}^{p_n}\left[\frac{\partial^2 Q_k(\boldsymbol{\beta}_0)}{\partial\beta_i\partial\beta_j} - E\left\{\frac{\partial^2 Q_k(\boldsymbol{\beta}_0)}{\partial\beta_i\partial\beta_j}\right\}\right]^2\right)\\ &= O_p(p_n^4 n^{-1}).\end{aligned}$$

□

Proof of Theorem S4

Proof. The proof is similar as Theorem S1. It suffices to prove that for any $\epsilon > 0$, with probability at least $1 - \epsilon$, there exist a constant C_ϵ such that a local minimizer exists within the ball

$$\{\boldsymbol{\beta}_0 + p_n^{1/2}n^{-1/2}\boldsymbol{u} : \|\boldsymbol{u}\| < C_\epsilon\}.$$

We first notice that by D.2,

$$\begin{aligned}p_n^{\frac{3}{2}}n^{-\frac{3}{2}}\left\|\frac{\partial}{\partial\boldsymbol{\beta}}\{\boldsymbol{u}^T\ddot{Q}_k(\boldsymbol{\beta}^*)\boldsymbol{u}\}\boldsymbol{u}\right\| &= p_n^{\frac{3}{2}}n^{-\frac{3}{2}}\left\|\sum_{i,j,k=1}^{p_n}\frac{\partial^3 Q(\beta)}{\partial\beta_i\partial\beta_j\partial\beta_k}u_iu_ju_k\right\|\\ &\leq p_n^{\frac{3}{2}}n^{-\frac{3}{2}}\left\|\sum_{i,j,k=1}^{p_n}M^2\right\|^{1/2}\|\boldsymbol{u}\|^3\\ &= o_p(p_nn^{-1})\|\boldsymbol{u}\|^3.\end{aligned}$$

Thus, by Taylor expansion,

$$\begin{aligned}S(\boldsymbol{\beta}_0 + p_n^{1/2}n^{-1/2}\boldsymbol{u}) - S(\boldsymbol{\beta}_0) \geq{}& \sum_{k=1}^K p_n^{1/2}n^{-1/2}\boldsymbol{u}^T\dot{Q}_k(\boldsymbol{\beta}_0) + \sum_{k=1}^K\frac{1}{2}p_nn^{-1}\boldsymbol{u}^T\ddot{Q}_k(\boldsymbol{\beta}_0)\boldsymbol{u}\\ &+ \sum_{j\in\mathcal{A}}p_n^{1/2}n^{-1/2}u_jp'_{\lambda_n}(|\beta_{0j}|)\text{sign}(|\beta_{0j}|) + \sum_{j\in\mathcal{A}}\frac{1}{2}p_nn^{-1}u_j^2p''_{\lambda_n}(|\beta_{0j}|) + o_p(p_nn^{-1})\\ ={}& I_{11} + I_{12} + I_{21} + I_{22}.\end{aligned}$$

By D.1, $\|I_{11}\| = O_p(p_n n^{-1})\|u\|$.
From Lemma S3,

$$I_{12} = \sum_{k=1}^{K} \frac{1}{2} p_n n^{-1} \boldsymbol{u}^T \left[\ddot{Q}_k(\boldsymbol{\beta}_0) - E\{\ddot{Q}_k(\boldsymbol{\beta}_0)\}\right] \boldsymbol{u} + \sum_{k=1}^{K} \frac{1}{2} p_n n^{-1} \boldsymbol{u}^T \left[E\{\ddot{Q}_k(\boldsymbol{\beta}_0)\}\right] \boldsymbol{u}$$
$$= \sum_{k=1}^{K} \frac{1}{2} p_n n^{-1} \boldsymbol{u}^T \left[E\{\ddot{Q}_k(\boldsymbol{\beta}_0)\}\right] \boldsymbol{u} + \frac{1}{2} o_p(p_n n^{-1})\|\boldsymbol{u}\|^2.$$

$$|I_{21}| \leq \sum_{j\in\mathcal{A}} |p_n^{1/2} n^{-1/2} u_j p'_{\lambda_n}(|\beta_{0j}|)\text{sign}(\beta_{0j})| \leq \max_{j\in\mathcal{A}}\{p'_{\lambda_n}(\beta_{0j})\} \sum_{j\in\mathcal{A}} |p_n^{1/2} n^{-1/2} u_j| \leq o_p(p_n n^{-1})\|\boldsymbol{u}\|$$

and

$$I_{22} \leq \max_{j\in\mathcal{A}}\{p''_{\lambda_n}(|\beta_{0j}|)\} \frac{1}{2} p_n n^{-1} \|\boldsymbol{u}\|^2 = o_p(p_n n^{-1})\|\boldsymbol{u}\|^2.$$

By choosing sufficient large $\|\boldsymbol{u}\|$, $I_{11} + I_{12} + I_{21} + I_{22}$ will be dominated by I_{12}, which is positive. Thus, $S(\boldsymbol{\beta}_0 + p_n^{1/2} n^{-1/2} \boldsymbol{u}) - S(\boldsymbol{\beta}_0) > 0$.

□
